# Analyses of rRNA gene chromatin in cell cycle arrested *Saccharomyces cerevisiae* cells

**DOI:** 10.1016/j.dib.2019.104083

**Published:** 2019-05-31

**Authors:** Audrey Paillé, Romain Charton, Alexia Muguet, Joachim Griesenbeck, Michael J. Smerdon, Antonio Conconi

**Affiliations:** a Département de Microbiologie et Infectiologie, Faculté de Médecine, Université de Sherbrooke, Sherbrooke, QC J1H 5N4, Canada; b Institut für Biochemie, Genetik und Mikrobiologie, Universität Regensburg, 93053 Regensburg, Germany; c Biochemistry and Biophysics, School of Molecular Biosciences, Washington State University, Pullman, WA 99164-7520, United States

**Keywords:** Alpha-factor, Chromatin, Hydroxyurea, Psoralen crosslinking, rRNA genes, RNA polymerase I

## Abstract

The existence of two chromatin structures in the rDNA locus was previously demonstrated for a large variety of organisms, ranging from yeast to human. In yeast there are about 150–200 rRNA genes organized in tandem repeats. Almost half of them are transcribed and largely depleted of nucleosomes (active/open), the other half is not transcribed and is assembled in regular arrays of nucleosomes (inactive/closed). It is proposed that RNA polymerase-I (RNAPI) transcription-elongation removes nucleosomes from closed rRNA genes (opening), and that soon after DNA replication there is deposition of nucleosomes on the open rRNA genes (closing). In G1 arrested cells, nearly all rRNA genes are depleted of nucleosomes, but most of them are not transcribed (inactive/open). In relation to the research article by *Charton* et al. (Mutat. Res.), the data presented here are on the hydroxyurea concentration-dependent inhibition of yeast culture growth, on cell cycle arrest before completion of genome replication, and on the opening of rRNA gene chromatin. As comparison, data are presented for yeast arrested in the G1-phase of the cell cycle by the pheromone α-factor.

**Table:** Specifications table

Subject area	*Biology*
More specific subject area	*Genetics, genomics, molecular biology (General)*
Type of data	*Graphs, figures*
How data was acquired	*Yeast treatments with HU or α-factor, cellular and biochemical analyses were done using standard techniques.*
Data format	*Raw data and analyzed data*
Experimental factors	*Saccharomyces cerevisiae strains JS311, JS306-A190MNbar1Δ.*
Experimental features	*Hydroxyurea and α-factor added to yeast cultures. Yeast growth curves obtained with a PowerWave Microplate scanning spectrophotometer. Cell cycle arrest followed by flow cytometry with Cytoflex B3-R1-V0. Psoralen photo-crosslinking of isolated nuclei from JS311 and JS306-A190MNbar1Δ. Southern blots of total DNA isolated from JS311 and JS306-A190MNbar1Δ strains, and digested with restriction enzymes.*
Data source location	*Dpt. of Microbiology and Infectiology, Faculty of Medicine, University of Sherbrooke, Sherbrooke, Canada.*
Data accessibility	*Data are in both, this article, in the related research article and in Mendeley database.*
Related research article	*Charton, R., Muguet, A., Griesenbeck, J., Smerdon, M.J., Conconi, A. In yeast cells arrested at the early S-phase by hydroxyurea, rRNA gene promoters and chromatin are poised for transcription while rRNA synthesis is compromised. Mutat. Res., 815 (2019) 20–29.*

**Table:** 

**Value of the data** • Yeast is an appropriate model organism to investigate the biochemistry of cell responses to genotoxic and cytotoxic compounds, like the chemotherapeutic agent hydroxyurea (HU). The data presented here on the effects of increasing doses of HU on yeast cultures will be useful to studies on the HU cellular responses. • There are nucleosomes on non-transcribed rRNA genes whereas there are few, if any, nucleosomes on transcribed rRNA genes. In both, G1 arrested cells by α-factor and early-S arrested cells by HU most rRNA genes are largely depleted of nucleosomes, despite not being transcribed. These data could help identifying the mechanisms that shape the chromatin of rRNA genes. • Because opening of rRNA gene chromatin and RNAPI transcription are coupled, the biochemical assays reported here could be applied to investigate the regulation of rRNA gene expression in the context of chromatin.

## 1 Data

Hydroxyurea (HU) arrests cell division by inhibiting DNA replication [1], [2]. The related research article describes that 200 mM HU arrests yeast (*Saccharomyces cerevisiae*) in the G1/early S-phase, and that the arrested cells have most rRNA genes in the open (non-nucleosomal) conformation [3]. Those results support a model proposing that there is deposition of nucleosomes on rRNA genes (closing) only after the passage of the replication fork through the rDNA locus [3 and references therein]. The data presented here describe the outcomes of treatments using lower concentrations of HU; 50 and 100 mM. Data are shown for the inhibition of culture growth, for cell cycle arrest and for the partition of rRNA genes between the two forms of chromatin. As comparison, data are also presented for yeast that are arrested in the G1-phase by the pheromone α-factor (α-F) [4].

**Fig. 1:**
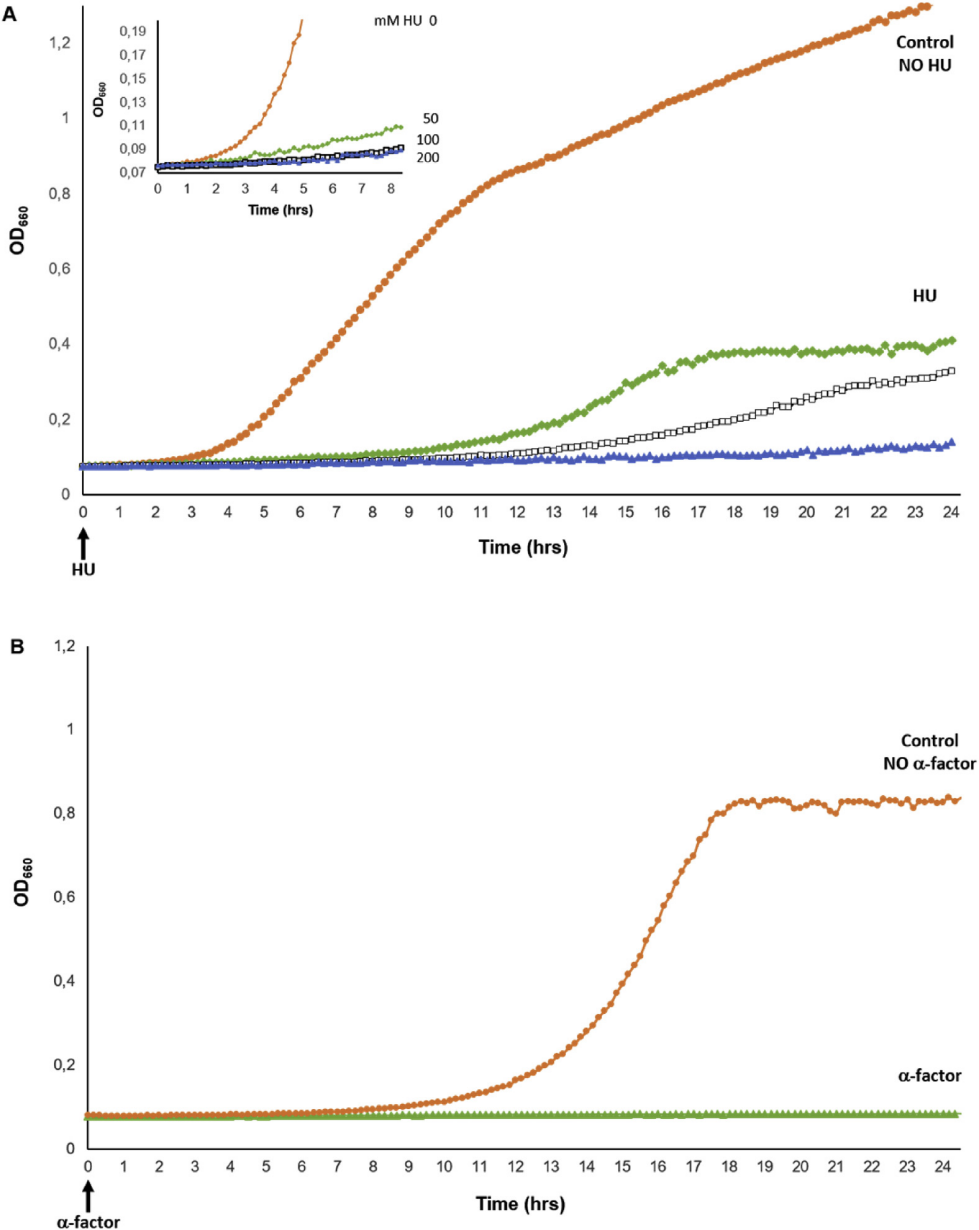
Yeast culture growths. (A) Yeast *JS311* were unexposed (doubling time of about 110 min; circles, orange) or exposed to 50 mM HU (diamonds, green), 100 mM HU (squares, black) and 200 mM HU (triangles, blue). Optical densities (OD_660nm_) were automatically recorded every 10 min. Arrow indicates the time 0 when HU was added to the cultures. Inset: enlargement of the y-axis scale. (B) Yeast *JS306-A1*90MN *bar1*Δ were mock treated or incubated with 50 ng/ml of α-F and measurements were taken as described in (A). Data represent the average of 3 independent biological replicates, each of which are the average of 4 technical replicates (https://doi.org/10.17632/3347b932g3.1).

Yeast cultures were mock treated or incubated with three different concentrations of HU. Cell growth was monitored over a period of 24 h by automated recording of optical densities [5], [6] (Fig. 1A). Measurements show that 50 and 100 mM HU significantly impair, but do not fully inhibit, culture growth. In contrast, even after 24 h, no significant increase in the optical density is observed for yeast culture that are incubated with 200 mM HU. Similarly, yeast cultures that were incubated in the presence of 50 ng/ml of the pheromone α-F do not grow for up to 24 h (Fig. 1B).

**Fig. 2:**
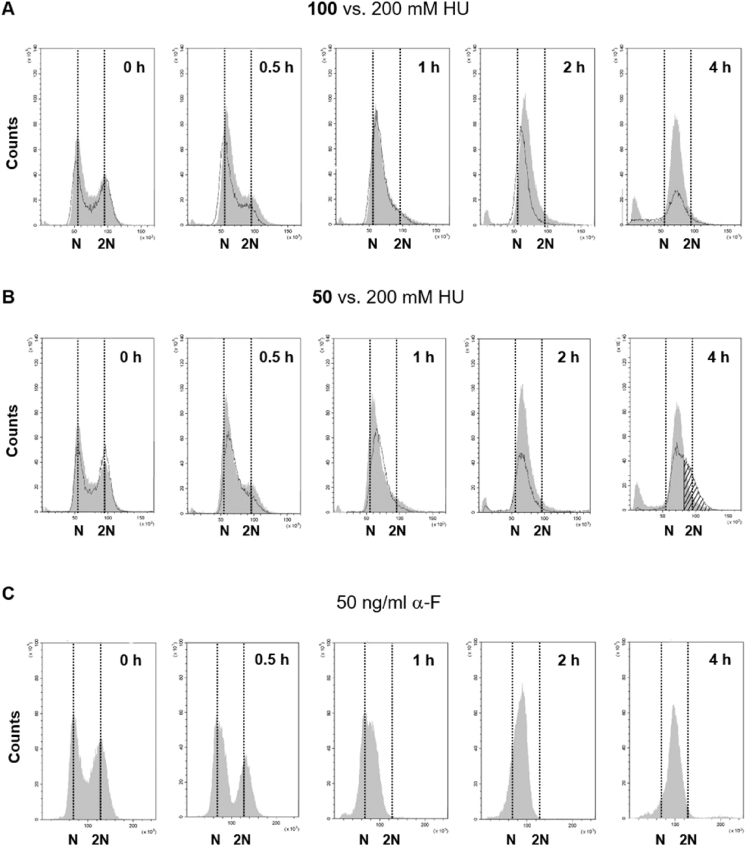
Analyses of cell cycle arrest by flow cytometry (FACS). Aliquots of yeast cells (*JS311*) exposed to 50, 100 and 200 mM HU, and aliquots of yeast cells (*JS306-A1*90MN *bar1*Δ) exposed to 50 ng/ml α-F were prepared for FACS. (A) Data for cells exposed to 100 mM (black curves) and 200 mM (grey curves) HU for the indicated time in hours. (B) Data for cells exposed to 50 mM (black curves) and 200 mM (grey curves) HU for the indicated time in hours. (C) Data for cells exposed to 50 ng/ml α-F for the indicated time in hours. Analyses of cell cycle arrest by FACS for 50 mM, 100 mM and 200 mM HU treatments were done in 4, 4 and 3 biological replicates, respectively. Analyses of cell cycle arrest by FACS for the 50 ng/ml α-F treatment were done in 2 biological replicates (raw data of Fig. 2; https://doi.org/10.17632/xvg7txzzkj.1).

Cell cycle arrest of yeast was monitored by flow cytometry (FACS). After 1–2 h incubation in the presence of 200 or 100 mM HU, most cells are synchronized at the G1/early S-phase (Fig. 2A; compare grey and black curves). Similar cell synchronization is observed during the first 2 h in the presence of 50 mM HU (Fig. 2B; compare grey and black curves). However, after 4 h incubation about 40% of the cell population appears to have duplicated at least part of the genomic DNA (Fig. 2B; stripped area). As comparison, yeast cells were synchronized by adding α-F to the media (Fig. 2C).

**Fig. 3:**
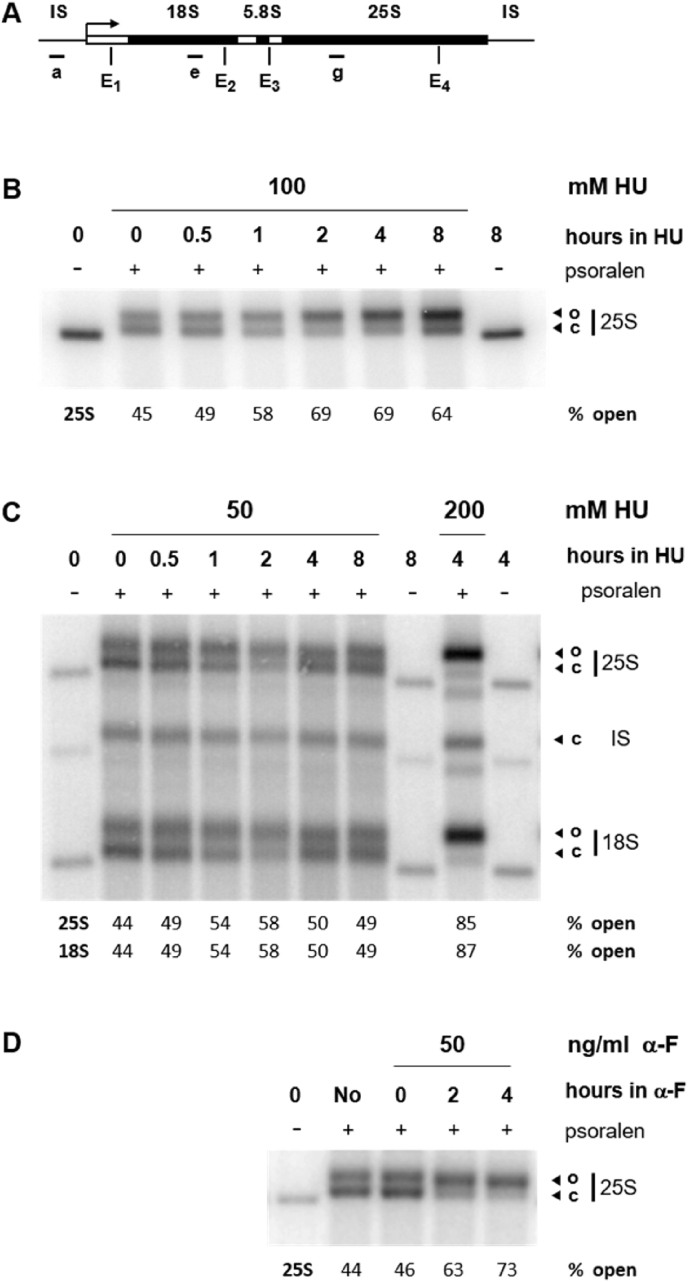
Psoralen photo-crosslinking of rRNA gene chromatin. Nuclei suspensions prepared from cells that were exposed to 50, 100 and 200 mM HU, to 50 ng/ml α-F or that were untreated (No α-F), were photo-crosslinked with psoralen. After DNA isolation and EcoRI digestion, the fragmented DNA was separated by native agarose gel electrophoresis. (A) Map of the coding region for the 18S, 5.8S and 25S mature rRNAs; IS corresponds to the intergenic spacer between rRNA genes that is largely covered with nucleosomes [8], [9]. The arrow points to initiation and direction of transcription, E_1_ to E_4_ show the approximate positions of the 4 EcoRI restriction sites; ‘a’, ‘d’ and ‘g’ represent the positions of end-labeled oligonucleotides that were used as probes (their sequences are specified in Tab. S2 of the related research article [3]). (B) Representative Southern blot illustrating the rRNA gene chromatin in 100 mM HU treated cells. After addition of HU, yeast cultures were incubated for 0–8 h. The respective nuclei suspensions were crosslinked with psoralen (+). As control, nuclei from the 0 and 8 h samples were not photo-crosslinked (−). After blotting, the filter membranes were hybridized with probe ‘g’. Symbols are (o) for open and (c) for closed rRNA gene chromatin. Quantifications of open chromatin in the 25S region are given as percent of total (open plus closed) chromatin. Similar results were obtained in 2 biological replicates. (C) Representative Southern blot illustrating the rRNA gene chromatin in 50 mM HU treated cells. The experiments were done as described in (B), except that all probes (‘a’, ‘d’, ‘g’) were added to the hybridization solution. To evaluate changes in the proportion of closed vs. open rRNA gene chromatin during treatment with 50 mM HU, nuclei were also isolated from yeast that were treated for 4 h in 200 mM HU and crosslinked with psoralen, as done in [3]. Symbols are as described in (B). Quantifications of open chromatin in the 25S and 18S regions are given as percent of total (open plus closed) chromatin. Similar results were obtained in 2 biological replicates. (D) Representative Southern blot illustrating the rRNA gene chromatin in untreated (No α-F) and α-F treated cells. After addition of α-F, yeast cultures were incubated for 0, 2 and 4 h. The experiments were done as described in (B), symbols and quantifications are as described above. Similar results were obtained in 3 biological replicates.

Psoralen photo-crosslinking is employed to analyze the proportion of closed to open rRNA genes [7]. In the following set of experiments the psoralen assay was applied to track the segregation of the two structures of rRNA genes in HU and α-F treated cells, or in control cells (No α-F) (Fig. 3). Yeast incubated with 100 mM HU for up to 8 h show a gradual shift in the proportion of the two forms of chromatin, from about 55% closed and 45% open at 0 h, to about 69% open between 2 and 8 h incubation (Fig. 3B; 25S). Similar data were found for yeast cultured in media containing 200 mM HU [3]. Opening of rRNA gene chromatin also takes place during the first 2 h in yeast treated with 50 mM HU, namely; 54% and 58% of the rRNA genes are open after 1 and 2 h incubation, respectively (Fig. 3C, 50 mM HU). However, in yeast treated with 50 mM HU closing of rRNA genes starts at 4 h incubation (50% closed and 50% open). As control, yeast were treated with 200 mM HU for 4 h, because at this treating condition yeast have a large proportion of open rRNA genes [3] (∼85%: Fig. 3C, 200 mM HU; 25S). Noteworthy, the intergenic spacer is covered with nucleosomes since it is largely not transcribed (Fig. 3A, IS) and, consequently, its structure does not change when cells are incubated with 50 or 200 mM HU (Fig. 3C; IS, c). For comparison, changes in the proportion of the two forms of rRNA gene chromatin were tracked in cells synchronized by α-F. Like for yeast arrested in 200 mM HU for over 2 h [3], α-F treated yeast have most rRNA genes in the open form (63–73%: Fig. 3D; 25S), as previously described [8].

## 2 Experimental design, materials, and methods

### 2.1 HU treatments, and monitoring of yeast culture growths by automatic measurements of optical densities

The *JS311* and *JS306-A190MNbar1*Δ strains [3], [10] were cultured in yeast extract-peptone-dextrose supplemented with adenine (Y_A_PD). Each well of a 96-well microplate (non-coated polystyrene) was prepared with 95 μl of Y_A_PD with 0, 50, 100, 200 mM HU or 50 ng/ml α-F. Subsequently, 5 μl of yeast cultures (∼8.10^6^ cells/ml) were added to each well and cell growth was monitored with a PowerWave microplate scanning spectrophotometer (Bio-Tek). The optical densities were automatically recorded using KC4 microplate data analysis software (Bio-Tek) [5], [6], [11], with readings taken every 10 min for 24 h.

### 2.2 Cell cycle arrest in early-S, or in G1 phase, and FACS analyses

Yeast were grown exponentially (∼1.2 × 10^7^ cells/ml) and treated with HU to the final concentrations of 50, 100 and 200 mM. After the indicated times, samples of cells were prepared for flow cytometry (FACS) as described in [11]. Alternatively, α-F was added to the yeast cultures at the final concentration of 50 ng/ml. At the indicated time, samples were collected and prepared for FACS. Measurements were made with a Cytoflex B3-R1-V0 cytometer (Beckman Coulter) and the data were analyzed with the CytExpert 2.3 software (Beckman Coulter).

### 2.3 Psoralen crosslinking, DNA extraction, agarose gel-electrophoresis and southern blot

Psoralen crosslinking of crude nuclei was done as previously described [9], [12]. Briefly, 10 μl of psoralen (4,5’,8-trimethylpsoralen, Sigma; 200 μg/ml of ethanol) was added to the well of a 24-multiwell plate containing 200 μl of nuclei (corresponding to ∼3.3 × 10^7^ cells). After 15 min incubation on ice and in the dark, nuclei were irradiated for 10 min, on ice, with a UVA lamp (UVP, Model TFL-40, Upland, CA91786 USA) that was placed at a distance of 15 cm. Addition of psoralen and photo-crosslinking was repeated 3 more times. After DNA extraction, agarose gel-electrophoresis and Southern blot, hybridization of the filter membranes were done as described in [9].

## References

[1] McGann P.T., Ware R.E. (2011). Hydroxyurea for sickle cell anemia: what have we learned and what questions still remain?. Curr. Opin. Hematol..

[2] da Fonseca M.A., Casamassimo P. (1997). Old drugs, new uses. Pediatr. Dent..

[3] Charton R., Muguet A., Griesenbeck J., Smerdon M.J., Conconi A. (2019). In yeast cells arrested at the early S-phase by hydroxyurea, rRNA gene promoters and chromatin are poised for transcription while rRNA synthesis is compromised. Mutat. Res..

[4] Bardwell L. (2004). A walk-through of the yeast mating pheromone response pathway. Peptides.

[5] Toussaint M., Levasseur G., Gervais-Bird J., Wellinger R.J., Abou Elela S., Conconi A. (2006). A high-throughput method to measure the sensitivity of yeast cells to genotoxic agents in liquid cultures. Mutat. Res..

[6] Toussaint M., Conconi A. (2006). High-throughput and sensitive assay to measure yeast cell growth: a bench protocol for testing genotoxic agents. Nat. Protoc..

[7] Toussaint M., Levasseur G., Tremblay M., Paquette M., Conconi A. (2005). Psoralen photo-crosslinking: a useful tool to study the structure of rDNA chromatin. Biochem. Cell Biol..

[8] Wittner M., Hamperl S., Stöckl U., Seufert W., Tschochner H., Milkereit P., Griesenbeck J. (2011). Establishment and maintenance of alternative chromatin states at a multicopy gene locus. Cell.

[9] Tremblay M., Charton R., Wittner M., Levasseur G., Griesenbeck J., Conconi A. (2013). UV light-induced DNA lesions cause dissociation of yeast RNA polymerase-I and establishment of a specialized chromatin structure at rRNA genes. Nucleic Acids Res..

[10] Smith J.S., Caputo E., Boeke J.D. (1999). A genetic screen for ribosomal DNA silencing defects identifies multiple DNA replication and chromatin-modulating factors. Mol. Cell. Biol..

[11] Toussaint M., Wellinger R.J., Conconi A. (2010). Differential participation of homologous recombination and nucleotide excision repair in yeast survival to ultraviolet light radiation. Mutat. Res..

[12] Conconi A., Widmer R.M., Koller T., Sogo J. (1989). Two different chromatin structures coexist in ribosomal RNA genes throughout the cell cycle. Cell.

